# Amino acid content of selected plant, algae and insect species: a search for alternative protein sources for use in pet foods*

**DOI:** 10.1017/jns.2014.33

**Published:** 2014-09-30

**Authors:** Sarah McCusker, Preston R. Buff, Zengshou Yu, Andrea J. Fascetti

**Affiliations:** 1 Department of Molecular Biosciences, School of Veterinary Medicine, University of California, Davis, CA, 95616, USA; 2 The Nutro Company, Franklin, TN 37067, USA

**Keywords:** Amino acids, Protein, Taurine, Canine nutrition, Feline nutrition, CP, crude protein, EAA, essential amino acids, MR, minimal requirements

## Abstract

In response to global economic duress and heightened consumer awareness of nutrition and health, sustainable and natural ingredients are in demand. Identification of alternative sources of nitrogen and amino acids, including taurine, may help meet dietary requirements while fostering sustainability and natural feeding approaches. Twenty plants, eighteen marine algae and five insect species were analysed. All samples were freeze-dried, hydrolysed and filtered prior to amino acid analysis. Samples for amino acids were analysed in duplicate and averaged. Nitrogen was analysed and crude protein (CP) determined by calculation. With the exception of taurine concentration in soldier fly larvae, all insects exceeded both the National Research Council's canine and feline minimal requirements (MR) for growth of all essential amino acids (EAA) and CP. Although some plants and marine algal species exceeded the canine and feline MR for growth for EAA and CP, only very low concentrations of taurine were found in plants. Taurine concentration in insects was variable but high, with the greatest concentration found in ants (6·42 mg/g DM) and adult flesh flies (3·33 mg/g DM). Taurine was also high in some macroalgae, especially the red algal species: *Mazaella* spp. (4·11 mg/g DM), *Porphyra* spp. (1·22 mg/g DM) and *Chondracanthus* spp. (6·28 mg/g DM). Preliminary results suggest that insects and some marine algal species may be practical alternatives to traditional protein and supplemental taurine sources in pet foods. Safety, bioavailability, palatability and source variability of alternative items as food ingredients should be investigated prior to incorporation into canine and feline diets.

Pet ownership brings many physiological and psychological benefits, and approximately one-third of households in the USA own a dog or cat^(^^1^^)^. That translates into approximately 70 million dogs and 74·1 million cats^(^^2^^)^. However, the sustainability of owning a pet has been a topic of public discussion recently, with a particular focus on nutrition.

Protein is the most expensive macronutrient in ecologic and economic terms and therefore the one requiring the most attention with respect to sustainability^(^^3^^)^. Protein is required for two reasons; to provide essential amino acids (EAA) that cannot be synthesised by dogs and cats from their diet, and to provide dispensable amino acids that in turn provide nitrogen and carbon for the synthesis of other dispensable amino acids, gluconeogenesis and energy. Dogs and cats require nitrogen and ten EAA, arginine, histidine, isoleucine, leucine, lysine, methionine, phenylalanine, threonine, tryptophan and valine. In addition, cats have a dietary requirement for taurine^(^^4^^)^.

Taurine is a sulphur-containing amino acid found in highest concentration within cardiac and skeletal muscle^(^^5^^,^^6^^)^. It has been documented to a lesser extent in algae, bacteria, fungi and some higher plants^(^^7^^–^^10^^)^. This free amino acid has a role in a variety of physiologic processes in animals, including conjugation of bile acids, maintaining normal retinal and myocardial function, osmoregulation and modulation of calcium flux within cells as well as reproduction and immune response^(^^5^^,^^6^^)^. Low blood or plasma taurine concentrations in strict carnivores have been directly linked to retinal degeneration and cardiomyopathy^(^^11^^–^^13^^)^, making taurine an EAA for cats^(^^4^^)^. Therefore, the minimum dietary inclusion concentrations of taurine in cat foods have been established by the Association of American Feed Control Officials (AAFCO) at 0·1–0·20 % (DM)^(^^14^^)^ and The European Pet Food Industry Federation (FEDIAF) at 0·2–0·25 % (DM)^(^^15^^)^. With the exception of felids and other strict carnivores, taurine is not recognised as an essential nutrient in most species as it can be synthesised from adequate dietary methionine and cystine^(^^5^^,^^13^^)^. Because it is not considered nutritionally essential beyond the cat, inclusion levels in canine foods have not been regulated. However, recent documentation of links between low blood and plasma taurine concentrations and the onset of dilated cardiomyopathy in dogs have been reported in the scientific literature^(^^16^^–^^18^^)^. These findings have motivated pet food companies to supplement taurine at discretionary levels in some commercial foods, leading to a demand for not only high-quality protein ingredients, but also additional sources of taurine.

The quality of ingredients and the inclusion of animal-based protein sources in pet foods are very important factors pet owners consider when selecting a diet^(^^19^^,^^20^^)^. This bias may put human subjects in direct competition with pets for many protein resources in the future. However, dogs and cats as well as human subjects need nutrients and not ingredients. Their nutrient requirements can be met through a variety, or often a combination, of animal or plant-derived protein sources.

In response to potential global economic duress and heightened consumer awareness regarding issues of nutrition and health, sustainable, and natural or organic ingredients are more in demand. Identification of additional sources of nitrogen and amino acids, including taurine, will help meet the dietary requirements of cats and dogs, lessening the competition for food resources. Therefore, our objectives were to report nitrogen and amino acid profiles of various plants, algal and insect substrates to begin to identify potential new ingredients for use in canine and feline diets.

## Experimental methods

### Acquisition and preparation of plants, algae and insects

It was our goal to select readily accessible, prolific plant species that can be cultivated and harvested at a rate conducive to production demands. We obtained produce items from local vendors. These items were rinsed then homogenised with a food processor. Marine algae samples were obtained from the UC Davis Bodega Bay Marine Laboratory (BML), Bodega Bay, CA, USA in February 2012. Holdfasts were removed and algae specimens were rinsed with deionised water and patted dry with paper towels. Cockroaches (*Periplaneta americana*), duckweed (*Lemna* spp.), blow fly larvae and adults (*Sarcophaga bullata*), and ants (*Pogonomyrmex occidentalis*) were purchased from Carolina Biological Supply Company (Carolina.com). Soldier fly larvae (*Hermetia illucens*) were purchased from Josh's Frogs (joshsfrogs.com). Live insects were fasted to empty GI tract of ingesta. All samples were stored frozen at −80°C prior to lyophilising using a standard vacuum coil freeze dryer (VirTis Freeze Mobile 25EL, SP Scientific).

### Amino acid and nitrogen analysis

After freeze-drying, all samples were ground to pass through a 2 mm screen (80 mesh). Roughly 10 mg of this ground sample was hydrolysed in a vacuumed-sealed glass ampule with 2 ml 6 m HCl at 115°C for 24 h. The hydrolysate was then dried with nitrogen gas and the resulting residue reconditioned with LiOH loading buffer. We filtered this solution using a 0·45 μm polytetrafluorethylene syringe filter, and from the filtrate, amino acid composition was determined using a norleucine standard and an automated amino acid analyser (HPLC, Biochrom 30, Biochrom Ltd) at the Amino Acid Laboratory at the University of California, Davis, CA, USA using previously published techniques^(^^9^^)^. Cystine and methionine were determined using the performic acid oxidation with acid hydrolysis, hydrobromic acid method^(^^21^^)^, and tryptophan using a previously described method^(^^22^^)^. DM of substrates was determined by placing a known amount of sample in a 100°C oven for 24 h, or until a stable weight was achieved. Samples for amino acid determination were analysed in duplicate for each item, averaged and reported on a DM basis. Owing to sample amount remaining, Trp was only analysed once. Nitrogen was analysed in the UC Davis Analytical Laboratory by combustion and crude protein (CP) was determined by calculation and reported on a DM basis^(^^23^^,^^24^^)^.

## Results

All insects exceeded both the National Research Council's canine and feline minimal requirements (MR) for growth of all EAA and CP (Tables 1 and 2). Some, but not all plants and marine algal species exceeded the canine and feline National Research Council's MR for growth for EAA and CP. No appreciable amount of taurine was found in any plants. Taurine concentration in insects was variable, but high, with the greatest concentration found in ants (6·42 mg/g DM) and adult flesh flies (3·33 mg/g DM). One species of green algae and many members of the group Phaeophyta did not yield high amounts of taurine. However, taurine was high in some red algal species including: *Mazaella* spp. (4·11 mg/g DM), *Porphyra* spp. (1·22 mg/g DM) and *Chondracanthus* spp. (6·28 mg/g DM). Dispensable amino acid concentrations for all plants, algae and insects analysed are reported in Tables 3 and 4 as supplemental material.

**Table 1.** Crude protein and essential amino acids of produce items

		Amino acid (mg/g DM)*											
	CP (% DM)†	Arg	His	Ile	Leu	Lys	Met	Phe	Tau	Thr	Trp	Val
Land cress (*Barbarea verna*)	45·5	17·72	6·39	11·20	20·51	18·74	1·40	15·18	0·04	12·83	4·63	15·74
Purple kale (*Brassica oleracea*)	27·6	5·5	3·62	4·78	7·32	8·33	2·34	4·33	0·02	6·16	1·60	8·25
Dandelion (*Taraxacum officinale*)	23·8	13·51	4·96	10·45	19·34	15·40	4·03	12·84	0·00	11·58	3·64	13·57
Red chard (*Beta vulgaris v. cicla*)	26·4	11·67	5·03	8·77	16·73	14·33	3·21	11·07	0·00	9·37	3·17	12·07
Okra (*Abelmoschus esculentus*)	15·3	8·69	2·60	4·57	8·33	9·00	2·01	4·88	0·02	5·65	1·60	6·29
Acai berry puree (*Euterpeoleracea*)	9·2	5·08	2·06	3·96	7·60	6·45	1·23	4·89	0·00	4·89	1·54	5·27
Prickly pear (*Opuntia ficus-indica* (fruits))	3·7	1·59	1·17	1·08	2·37	2·07	0·49	1·40	0·00	1·18	0·31	1·46
Pomegranate (*Punica granatum*)	7·6	6·33	1·53	1·78	3·62	1·84	0·79	2·12	0·00	1·87	0·95	2·12
Blueberry (*Vaccinium cyanococcus*)	4·7	4·12	0·69	1·05	2·14	1·96	0·44	1·25	0·00	1·22	0·56	1·40
White sapote (*Casimiroa edulis*)	3·6	1·56	0·77	1·18	2·80	2·41	0·45	1·46	0·00	1·50	0·45	1·58
Plum (*Prunus* spp.)	3·9	0·67	0·52	2·16	1·69	1·41	0·19	1·05	0·00	0·99	0·15	1·24
Beet greens (*Beta vulgaris v. conditiva*)	25·9	8·63	4·43	6·62	11·90	10·55	2·30	7·66	0·02	6·64	2·26	8·93
Beetroot (*Beta vulgaris v. conditiva*)	17·7	3·32	2·30	3·22	4·90	4·53	1·05	2·32	0·00	2·91	0·75	3·61
Cherimoya (*Annona cherimola*)	6·6	2·58	1·22	2·04	4·15	3·82	0·82	2·62	0·00	2·14	0·55	2·64
Jalepeno pepper (*Capsicum annum*)	14·7	6·59	2·72	5·13	8·93	9·90	2·08	6·36	0·01	5·97	1·21	6·91
Eggplant (*Solanum melongena*)	14	7·18	2·92	4·63	7·66	8·10	1·55	4·69	0·00	4·22	1·12	6·70
Taro root (*Colocasia esculenta*)	7·3	6·10	1·68	2·67	6·82	3·88	0·96	4·32	0·00	3·16	0·86	4·13
Brewer's yeast (*Saccharomyces cerevisiae*)	55·2	25·53	11·25	24·31	37·20	44·26	8·06	22·52	0·00	25·74	6·18	30·26
Duckweed (*Lemna* spp.)	24·8	11·12	3·43	6·72	15·26	11·54	3·13	9·85	0·00	8·33	3·10	9·05
Cactus fruit (*Ferocactus* spp.)	10·6	14·13	2·77	3·30	6·51	3·83	3·24	4·5	0·00	3·4	1·41	3·96
NRC MR Canine for growth	18·0	6·3	3·1	5·2	10·3	7·0	2·8	5·2	NR‡	6·5	1·8	5·4
NRC MR Feline for growth	18·0	7·7	2·6	4·3	10·2	6·8	3·5	4·0	0·32	5·2	1·3	5·1

CP, Crude protein; NRC MR, National Research Council Minimal Requirement; NR, not required.

*Sample for each amino acid determination analysed in duplicate and reported as a mean value, with the exception of Trp in which only one sample was analysed due to sample size.

†Crude protein expressed on a % (w/w) DM basis.

‡Taurine is not considered an essential amino acid in the dog.

**Table 2.** Crude protein and essential amino acids of selected marine macroalgae and insects

		Amino acid (mg/g DM)*											
	CP (% DM)†	Arg	His	Ile	Leu	Lys	Met	Phe	Tau	Thr	Trp	Val
Chlorophyta (green algae)												
*Ulva* spp.	28·5	16·00	4·52	9·26	16·76	11·61	4·47	11·67	0·01	14·34	4·55	16·32
Phaeophyta (brown algae)												
*Eisenia arborea*	26·2	17·35	9·94	8·88	14·20	18·02	4·12	9·66	0·07	11·92	1·76	12·66
*Hedophyllum sessile*	15·6	6·17	2·29	5·37	10·27	9·26	2·60	7·10	0·02	8·77	1·32	8·09
*Dictyoneurum californicum*	17·2	6·3 5	2·56	5·66	9·30	10·11	2·68	6·72	0·00	8·65	1·74	8·19
*Macrocystis* spp.	17·6	4·04	1·35	3·29	5·69	4·30	2·00	3·79	0·10	3·63	2·13	4·46
*Laminaria* spp.	14·0	5·42	2·21	4·34	8·39	9·97	2·17	5·48	0·02	7·01		6·68
*Fucus gardneri*	11·7	5·30	1·85	4·59	8·11	7·25	2·36	5·17	0·00	5·22	1·63	5·70
*Egregria menziessii*	14·9	6·69	2·33	5·89	10·06	11·66	2·60	7·02	0·02	7·58	1·65	8·52
*Pelvetropsis limitata*	11·4	4·29	1·61	3·76	6·67	6·30	1·93	4·38	0·01	4·56	1·42	4·91
*Lessoniopsis littoralis*	14·8	5·04	1·70	4·82	8·66	12·08	2·25	5·75	0·02	8·10	1·12	8·48
*Pterygophora californica*	11·4	3·40	1·63	3·23	5·77	7·25	1·58	3·79	0·00	4·84	1·04	5·00
Rhodophyta (red algae)												
*Mazzaella* spp.	17·5	11·76	2·45	6·88	11·68	10·09	3·44	7·37	4·11	6·93	1·39	8·90
*Palmaria hecatensis*	23·6	12·56	3·25	8·55	15·33	15·40	4·91	10·19	0·60	10·46	2·91	12·08
*Neorhodo melalarix*	21·2	9·12	3·05	6·96	11·18	16·66	3·73	9·70	0·10	8·19	1·31	8·79
*Porphyra* spp.	8·3	6·93	1·27	3·61	6·16	5·18	1·66	4·25	1·22	3·69	0·54	4·79
*Cryptopleura ruprechtiana*	27·9	16·81	7·73	8·80	14·50	23·00	4·15	11·92	0·01	13·04	2·90	13·16
*Chondracanthus* spp.	14·8	10·23	2·27	6·00	10·23	8·48	2·92	7·02	6·28	6·74	1·46	8·17
*Prionotis* spp.	36·9	19·39	4·10	13·84	20·84	20·91	9·34	16·66	0·03	15·02	3·39	17·25
Insects												
American cockroach (*Periplaneta americana*)	95·8	33·71	15·41	18·81	35·86	34·91	8·90	19·68	1·10	21·01	4·71	30·99
Flesh fly, larva (*Sarcophaga* (*Neobelliaria*) *bullata*)	65·8	35·69	23·47	27·40	44·86	56·14	15·96	40·62	0·92	27·15	8·93	35·46
Western harvester ant (*Pogonomyrmexoccidentalis*)	66·3	25·72	15·13	30·33	46·92	28·42	8·03	16·15	6·42	24·36	NA	37·94
Flesh fly, adult (*Sarcophaga* (*Neobelliaria*) *bullata*)	78·6	44·27	24·88	32·05	51·80	61·36	19·91	32·37	3·33	29·00	9·00	40·39
Black soldier fly, larva (*Hermetiaillucens*)	46·7	23·25	15·60	17·30	28·75	28·57	6·94	16·00	0·19	16·54	6·26	26·01
NRC MR Canine for growth	18·0	6·3	3·1	5·2	10·3	7·0	2·8	5·2	NR‡	6·5	1·8	5·4
NRC MR Feline for growth	18·0	7·7	2·6	4·3	10·2	6·8	3·5	4·0	0·32	5·2	1·3	5·1

CP, Crude protein; NRC MR, National Research Council Minimal Requirement; NR, not required; NA, not adequate sample for analysis.

*Sample for each amino acid determination analysed in duplicate and reported as a mean value, with the exception of Trp in which only one sample was analysed due to sample size.

†Crude protein expressed on a % (w/w) DM basis.

‡Taurine is not considered an essential amino acid in the dog.

## Discussion

In order to address the growing importance of protein sources in animal nutrition, we sought to identify alternative, sustainable sources of nitrogen and EAA. While the use of plant-derived proteins is common practice in commercial canine and feline diets, the investigation of plants as a source of taurine has either been overlooked or very difficult as indicated by the lack of reports in the literature. Other alternative sources of nitrogen and EAA such as insects, fungi, and algae have also been little studied. Identification of non-animal tissue sources of nitrogen and EAA, including taurine, will expand the substrates available to animal food manufacturers, enhancing flexibility within diet formulation. The potential economic benefit of propagating such sources compared to animal pools is a likely avenue to decrease production costs while supporting sustainability.

With the exception of taurine concentration in soldier fly larvae, all insects analysed in the present study yielded CP, EAA and taurine concentrations in excess of the National Research Council's MR for dogs and cats. Taurine ranged from 0·19 mg/g DM in soldier fly larvae to 6·42 mg/g DM in harvester ants. Similarly, Finke found insects to be a good source of nutrients, including taurine. Crickets (*Acheta domestics*) were highest among species analysed in that report, yielding 1·4 mg taurine/g for adults and 0·8 mg taurine/g for nymphs on an as is basis^(^^25^^)^. Although insects provide adequate amounts of EAA, acceptability as a pet food ingredient may be variable in different cultures. Additionally, insects feed on a variety of substrates, including plants that are known to be toxic to companion animals. The authors concluded that the safety and palatability of insects as food for dogs and cats should be investigated prior to incorporation into commercial diets^(^^25^^)^.

Not all algal species analysed met or exceeded the National Research Council's MR for dogs and cats for CP or EAA. For alternative taurine sources, the most promising substrates in the present study were species of red algae, including *Chondracanthus* spp*., Mazaella* spp, and *Porphyra*s pp. all of which met or exceeded the AAFCO and FEDIAF feline requirementfortaurine (0·1 % DM) in extruded diets and the first two also exceeding the AAFCO and FEDIAF feline requirement for canned foods (0·2, 0·25 % DM, respectively). Of particular interest is the *Chondracanthus* specimen, which yielded an average taurine concentration of 6·3 mg/g DM, similar to concentrations reported for various seafood items (i.e. fish or clams), and the *Mazzaella* specimen, with similar taurine concentration to that of animal tissue^(^^9^^)^.Our findings are in agreement with another study that reported red algae contained higher concentrations of taurine (0·13 g/kg as is) compared to green (0·002 g/kg as is) or brown (0·01 g/kg as is) algae species^(^^7^^)^.

As with insects, further investigation is necessary before incorporating various species of algae into animal diets. Some species of brown algae have been shown to contain polysaccharides that have anti-pepsin activity *in vitro*, and could compromise overall protein digestibility of a diet^(^^26^^)^*.* It is possible that seaweed polysaccharides whose physiochemical properties have not been investigated also exhibit anti-nutritional characteristics, but more research is needed.

Similar to several previous investigations, standard grocery store items (fruits and vegetables) yielded no significant amounts of taurine. Contrary to other investigations that reported fruits from the *Opuntia* cultivars to contain taurine in the range of 0·07 to 0·57 g/kg on an as is basis^(^^8^^,^^10^^,^^27^^)^, the cactus fruit analysed in our study did not yield any taurine. While not specifically analysed in this preliminary study, prune juice concentrate obtained from local sources in Davis, CA has also been reported to contain taurine (0·02–0·16 g/kg as is), although source and method of processing yielded different results^(^^28^^)^. It has been notoriously difficult to identify plants that contain measurable amounts of taurine^(^^5^^,^^9^^,^^29^^)^, and often the amino acid is overlooked or underreported in analysis of plant matter.

Nutritional quality of plants and macroalgae have also been shown to vary with season, geographic location (soil nutrients), and environmental stress (drought, salinity, light exposure)^(^^30^^–^^33^^)^. In the case of the red seaweed, *Palmaria palmata,* which is native to Atlantic regions, there is variation in amino acid content from year to year and season to season, with the highest protein content found in specimens collected in the winter-spring period^(^^34^^)^. It is possible that amino acids in the substrates analysed in our lab also vary within the described parameters, thus explaining any differences in values obtained.

Using hydrolysis to analyse low concentrations of taurine is not without error. In his review of the physiological actions of taurine, Huxtable explains that certain molecules can coelute, leading to an exaggerated peak or false identification of taurine^(^^5^^)^. Another potential source contributing to error could be marine organisms not rinsed from samples upon initial processing.

Preliminary results suggest that insects and some marine algal species may be practical alternatives to more traditional protein and supplemental taurine sources in pet foods. Safety, bioavailability, palatability and source variability of alternative items as feed ingredients should be investigated prior to incorporation into canine and feline diets.

## Supplementary material

The following text to be added when online supplementary material given: The supplementary material for this article can be found at http://www.journals.cambridge.org/jns
